# Evolutionary Dynamics of Transposable Elements Following a Shared Polyploidization Event in the Tribe Andropogoneae

**DOI:** 10.1534/g3.120.401596

**Published:** 2020-09-28

**Authors:** Dhanushya Ramachandran, Michael R. McKain, Elizabeth A. Kellogg, Jennifer S. Hawkins

**Affiliations:** * Department of Biology, 53 Campus Drive, West Virginia University, Morgantown, WV 26506; † Donald Danforth Plant Science Center, 975 North Warson Rd., St. Louis, MO 63132; ‡ Department of Biological Sciences, 300 Hackberry Lane, The University of Alabama, Tuscaloosa, AL 35487

**Keywords:** adaptation, LTR retrotransposon, *copia* insertions, maize domestication, *Tripsacum dactyloides*

## Abstract

Both polyploidization and transposable element (TE) activity are known to be major drivers of plant genome evolution. Here, we utilize the *Zea-Tripsacum* clade to investigate TE activity and accumulation after a shared polyploidization event. Comparisons of TE evolutionary dynamics in various *Zea* and *Tripsacum* species, in addition to two closely related diploid species, *Urelytrum digitatum* and *Sorghum bicolor*, revealed variation in repeat content among all taxa included in the study. The repeat composition of *Urelytrum* is more similar to that of *Zea* and *Tripsacum* compared to *Sorghum*, despite the similarity in genome size with the latter. Although LTR-retrotransposons were abundant in all species, we observed an expansion of the *copia* superfamily, specifically in *Z. mays* and *T. dactyloides*, species that have adapted to more temperate environments. Additional analyses of the genomic distribution of these retroelements provided evidence of biased insertions near genes involved in various biological processes including plant development, defense, and macromolecule biosynthesis. Specifically, *copia* insertions in *Zea* and *T. dactyloides* were significantly enriched near genes involved in abiotic stress response, suggesting independent evolution post *Zea-Tripsacum* divergence. The lack of *copia* insertions near the orthologous genes in *S. bicolor* suggests that duplicate gene copies generated during polyploidization may offer novel neutral sites for TEs to insert, thereby providing an avenue for subfunctionalization via TE insertional mutagenesis.

Transposable element (TE) activation and accumulation generates significant genetic variation that can confer a range of effects on genome structure and function. As TEs carry ‘ready-to-use’ *cis*-elements, their insertions can impact gene regulation on a genome-wide scale by providing assorted regulatory elements to the adjacent genes. The new regulatory elements offered by inserted TEs can amplify and/or redistribute transcription factor binding sites, thereby creating new regulatory networks or even participate in re-wiring of pre-existing networks (Hènaff *et al.* 2014, Lavialle *et al.* 2013, Krupovic *et al.* 2014, Huang *et al.* 2016, Carmona *et al.* 2016, Joly-Lopez *et al.* 2016). Several empirical studies have demonstrated TE-induced phenotypic changes associated with domestication and/or diversification of cultivated plants, including rice, maize, wheat, soybean, melon, and palm (Xiao *et al.* 2008, Naito *et al.* 2009, Fernandez *et al.* 2010, Studer *et al.* 2011, Uchiyama *et al.* 2013, Sanseverino *et al.* 2015, Ong-Abdullah *et al.* 2015, Lu *et al.* 2017). Indeed, TE-related polymorphisms are responsible for phenotypic variation in many agronomically important crops, demonstrating their importance in creating the genetic variability that contributes to plant genome evolution.

Hybridization, polyploidy, and stress have been shown to correlate with transposable element movement (Steward *et al.* 2000, Kalendar *et al.* 2000, Madlung *et al.* 2002, Ungerer *et al.* 2006, Ito *et al.* 2011, Cavrak *et al.* 2014, Bardil *et al.* 2015, Guo *et al.* 2017). Flowering plants are known to tolerate hybridization and polyploidy, both of which have promoted species diversification (Payseur and Rieseberg, 2016, Soltis *et al.* 2016, Goulet *et al.* 2017). These phenomena result in TE mobilization, leading to local mutations and genome size changes (Liu and Wendel 2000; Josefsson *et al.* 2006; Ungerer *et al.* 2006; Kawakami *et al.* 2010; Parisod *et al.* 2010; Piednoël *et al.* 2013). Furthermore, such bursts of TE activity result in insertional polymorphisms, often with deleterious effects on genome function; however, these effects could be nullified or shielded via gene duplication in polyploid genomes. Although the relationship between TE mobility and hybridization and/or polyploidization is unclear, it is speculated that such TE reactivation in response to genomic stresses could be due to incompatible suppression machinery between the two donor genomes, or that unknown mechanisms are in place that reduce genomic methylation under general stress conditions (Ha *et al.* 2009, Yaakov and Kashkush 2012, An *et al.* 2014, Senerchia *et al.* 2014, Defraia and Slotkin 2014, Ågren *et al.* 2016).

Previous studies of polyploidy in *Zea* have revealed evidence for a whole genome duplication (WGD) event at or shortly after the origin of grasses (Paterson *et al.* 2004), followed by another, more recent, WGD in the history of *Zea* that promoted the origin of the *Zea-Tripsacum* clade (Estep *et al.* 2014; McKain *et al.* 2018). Diversifying from a common ancestral allotetraploid (n = 20), both the *Zea* (n = 10) and *Tripsacum* (n = 18) genomes responded differentially to the diploidization process (Swignová *et al.* 2004, Schnable *et al.* 2009, Schnable & Freeling 2011). In addition to these chromosomal rearrangements, there is also evidence for retrotransposon invasion post divergence in both *Zea* and *Tripsacum* (Gaut *et al.* 2000). Hence, being divergent descendants of a common allopolyploid ancestor, the *Zea-Tripsacum* clade is a good model system to understand various evolutionary processes including the contribution of TEs to polyploidy, rediploidization, and species diversification.

Here, we describe TE activity and contribution to genome diversity in the *Zea-Tripsacum* clade that has undergone a recent shared polyploidization event (Figure 1A). We included two diploid relatives, *Urelytrum digitatum* and *Sorghum bicolor*, which provide an opportunity to explore TE-associated evolutionary events induced by hybridization and genome doubling. By using clustering analysis, we have characterized the repetitive landscape in three *Zea* and three *Tripsacum* species (post allopolyploidization) and compared these results with that of the diploid relatives (pre-allopolyploidization). Our findings suggest recent and post-divergence activity of TEs in *Zea* and *Tripsacum* with lineage-specific expansions of *copia* elements specifically in *Z. mays* and *T. dactyloides*. Additionally, distributional analyses of newly expanded LTR-retrotransposons show insertional biases near genes involved in anatomical structure development, multicellular organismal processes, development, stress response, and defense.

**Figure 1:**
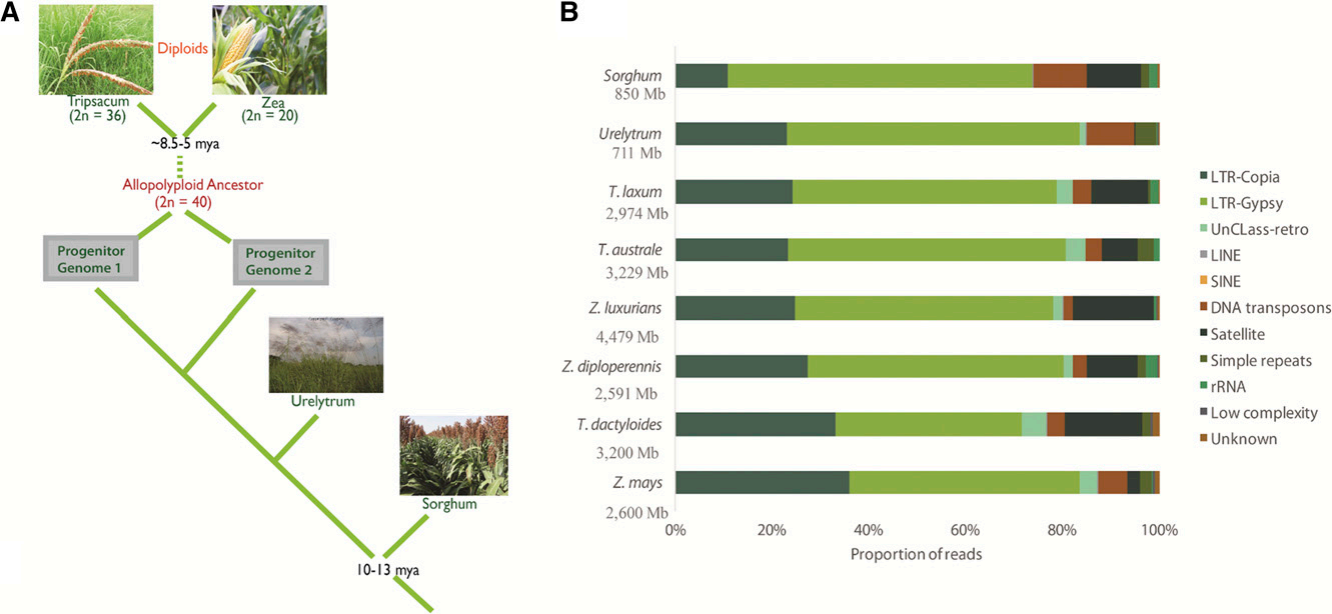
A. Evolutionary relationships of selected grass species, indicating polyploidization and species divergence. B. Proportional repeat composition. Genome size in Mb shown for each species in the y-axis. An expansion of *copia* families is observed in both *Z. mays* and *T. dactyloides* compared to related sister species. *Sorghum* displays a predominance of *gypsy* elements with a low level accumulation of *copia* families compared to *Zea-Tripsacum-Urelytrum* genomes.

## Materials and methods

### Plant material sources and Illumina sequencing of DNA

The following eight panicoid grasses were used in this study: *Zea mays* L., *Z. diploperennis* Iltis, Doebley & R. Guzmán, *Z. luxurians* (Durieu & Asch.) R.M. Bird, *Tripsacum dactyloides* (L.) L., *T. laxum* Nash, *T. australe* Cutler & E.S. Anderson, *Urelytrum digitatum* K.Schum., and *Sorghum bicolor* (L.) Moench. Short-read sequence data for *Zea mays* (SRS291653), *Zea luxurians* (SRR088692), *Tripsacum dactyloides* (SRS302460), and *Sorghum bicolor* (SRR5271056) were downloaded from the NCBI short read archive (Chia *et al.* 2012, Tenaillon *et al.* 2011, McCormick *et al.* 2018). Genomic short-read sequences of *Zea diploperennis*, *Tripsacum laxum*, *Tripsacum australe*, and *Urelytrum digitatum* were obtained from Dr. Elizabeth Kellogg, Donald Danforth Plant Science Center, St. Louis, Missouri and are deposited in (BioProject PRJNA664114).

### Identification of TE families

Sequences were quality trimmed with Trimmomatic v0.33 (Bolger *et al.* 2014) using a sliding window of 4:25 and minimum length of 50 bp. Graph-based clustering of quality-trimmed reads was performed with RepeatExplorer, a pipeline designed to identify repeats from NGS reads (Novák *et al.* 2013). RepeatExplorer employs a clustering algorithm that quantifies similarities between all sequence reads and produces a graph that consists of nodes (sequence reads) and edges (connecting overlapping reads). Nodes are frequently connected to one another if they pass a threshold of 90% similarity over at least 55% of the sequence length, representing individual repetitive families.

Species-specific clustering analysis provides information regarding repeat quantities by reporting the number of reads per cluster, which can then be used to estimate the genome space occupied by each particular repeat, *i.e.*, (total length of each cluster (in Mb) x genome size (in Mb)) / total length of all clusters (in Mb) (Macas *et al.* 2015, Kelly *et al.* 2015, Ramachandran and Hawkins 2016). For species-specific clustering, three million reads (approximately 0.2x to 0.5x genome coverage) were sub-sampled from each dataset and processed to the format required by RepeatExplorer. Subsequently, all of the processed reads from all species were concatenated into one combined dataset, and the RepeatExplorer clustering was repeated in order to facilitate comparative analysis. All clusters were annotated using the Viridiplantae RepeatMasker library (<http://www.repeatmasker.org>) and categorized into repeat families. A plot representing interactions between repeat clusters among species was created using UpSetR (Lex *et al.* 2014).

### Quantitative analysis of TE activity using molecular clock analysis

To estimate the timing of TE activity in each lineage, species-specific LTR sequences were extracted from each LTR-retroelement cluster. These species-specific reads were assembled using the Geneious *de novo* assembler to obtain a consensus sequence (Kearse *et al.* 2012). A grass-specific database was then used to extract LTRs from each consensus contig (blastn, e-value 1e-10, 85% identity). The best match for each species was chosen and the corresponding hit region was extracted using BEDTools v2.17.0 (Quinlan and Hall 2010).

To calculate LTR divergence (a rough measurement to estimate the age of a specific retrotransposon family), reads that were used for *de novo* assembly were mapped to the consensus LTR sequence using the Geneious reference genome assembler. To compare the LTRs of each species-specific repeat cluster, the percent identity was derived from the reference alignment by mapping each read to its respective LTR consensus sequence. Using a grass specific transposable element substitution rate of 1.3 × 10^−8^ per site per year (Ma and Bennetzen 2004), we estimated the activity of each major TE family in each species.

### Genomic distribution of retroelements

Genome wide analyses for insertional bias of retroelements near protein coding genes in maize and sorghum were performed using the gene annotations for *Z. mays* (Zmays_284_5b+) and *S. bicolor* (Sbicolor_313_v3.1), downloaded from Phytozome v12.1 (Goodstein *et al.* 2012). The maize TE annotation file (B73v4.TE.filtered.gff3) was downloaded from Gramene Release 62 (http://www.gramene.org) and sorghum retroelement annotations were retrieved from our previous work described in Ramachandran and Hawkins 2016. BEDtools closest (version 2.17.0; Quinlan and Hall 2010) was used to identify LTR-retrotransposon (*gypsy* and *copia*) insertions within 1-5 kb upstream of genes. Insertion dating was performed as described above (Ma and Bennetzen 2004). Further, for genes associated with upstream retrotransposon insertions, gene set enrichment analysis was performed in ShinyGO v0.6.1 (http://bioinformatics.sdstate.edu/go/; Ge *et al.* 2020).

As *Tripsacum* lacks a reference genome assembly, insertional bias analysis was performed using *Z. mays* reference gene annotations. *T. dactyloides* paired-end reads for which at least one read matched to our *gypsy* and *copia* clusters were identified using BLASTn v2.2.28+ (e-value = 1e-10, percent identity = 80). Matched reads were extracted and mapped to a library consisting of *copia* and *gypsy* contigs assembled by RepeatExplorer and to a filtered gene set containing the protein-coding genes from the *Z. mays* reference genome. Reference mapping of paired-end reads to the library was carried out using BWA aln v0.7.12 (Li and Durbin 2009) with the following optimized parameters: using the first 12 bases as a seed (-l 12), a maximum edit distance of four (-n 4), a maximum edit distance of two for the seed (-k 2), up to three gap openings (-o 3), a maximum of three gap extensions (-e 3), a mismatch penalty of two (-M 2), a gap opening penalty of six (-O 6), and a gap extension penalty of 3 (-E 3) (Mascagni *et al.* 2015). The results were used to generate a SAM file via the BWA “sampe” module, and then converted to a BAM file using SAMtools v1.9 (Li *et al.*, 2009). An LTR-retrotransposon was considered near a gene if one of the paired-end reads mapped to an LTR-retrotransposon and the other to a gene. The analysis was repeated for the remaining species included in the study (*Z. diploperennis*, *Z. luxurians*, *T. laxum*, *T. australe*, *U. digitatum*).

### Phylogenetic analysis of retroelement families

To assess the evolutionary relationships of the shared *gypsy* and *copia* families, the reverse transcriptase (RT) and integrase (INT) amino acid domains were used for phylogenetic analysis. RepeatExplorer clusters were filtered for LTR-*gypsy* and *copia* elements with RT and INT domain blastx hits. RT reads were extracted from each cluster using the blastx output file and placed in separate genome-specific files. The reads were assembled for each cluster using the Geneious *de novo* assembler (Kearse *et al.* 2012). The resulting contigs were then confirmed to contain reverse transcriptase domains using blastx against the Cores-RT database (Llorens *et al.* 2011). RT sequences were then combined into a final query file for further analysis. The same analysis was performed for INT reads using Cores-INT database (Llorens *et al.* 2011).

Rpstblastn (e-value = 1e-10) was performed for the sequence dataset against the Conserved Domain Database (Marchler-Bauer *et al.* 2015) to identify and extract conserved regions. The best hits for each sequence were extracted, and the filtered blast output was converted to three-column bed format with matching coordinates for each hit. BEDTools v2.170 (Quinlan and Hall 2010) was used to extract the conserved regions (∼540 bp for the RT domain and ∼340 bp for the INT domain). The correct open reading frame from each sequence was identified using ORFfinder. All amino acid sequences were globally aligned with MUSCLE v3.8.31 (Edgar 2004). Alignments were manually inspected and adjusted in Bioedit v7.3.5 (Hall 1999). The optimal model of amino acid substitution for each alignment was estimated using Prot-test v3.4.5 according to the Akaike Information Criterion (AIC) (Abascal *et al.* 2005). In all cases except RT-*copia*, the best model selected was LG+G (Le and Gascuel. 2008). Blosum62+G was chosen as the optimal model for RT-*copia* (Henikoff and Henikoff. 1992). Likelihood analyses with 1,000 bootstrap replicates were performed in RAxML v.8.2.9 (Stamatakis *et al.* 2008) using the best model for each alignment. Bayesian analysis of alignments was performed in MrBayes v3.2.6 using rates = gamma and the respective substitution model (Ronquist and Huelsenbeck 2003). Two independent MCMC runs of 10 million generations were performed, sampling each run every 1,000 generations until convergence, with the potential scale reduction factor close to 1. All trees were visualized using FigTree v1.4.0.

### Data availability

The authors affirm that all data necessary for confirming the conclusions of this article are represented fully within the article and its tables and figures. All sequence data are deposited as described in the Methods. Supplemental material available at figshare: https://doi.org/10.25387/g3.11954496.

## Results

### Single-species clustering analysis

To evaluate repeat content with respect to genome size, we performed a separate clustering analysis for each species. Individual clustering allows the maximum number of reads to assemble, which increases the accuracy of the repeat estimates. We estimated the quantities of each repeat family in the genome using the following equation: (total length of each cluster (in Mb) x genome size (in Mb)) / total length of all clusters (in Mb) (Macas *et al.* 2015, Kelly *et al.* 2015). The estimated repeat compositions are shown in Table 1.

**Table 1:** Global repeat composition (in Mb) of species with respect to genome size. Genome size (in Mb) for each species is given below each species name. Estimated repeat content (in Mb) for each repeat family is listed below using individual repeat clustering analysis. Bold text represents the most abundant families in each genome.

	*Z. mays* (2600 Mb)	*Z. dip* (2591 Mb)	*Z. lux* (4479 Mb)	*T. dact* (3200 Mb)	*T. aus* (3229 Mb)	*T. lax* (2974 Mb)	*Urelytrum* (711 Mb)	*Sorghum* (848 Mb)
LTR/*Copia*								
*ji*	444.02	362.72	519.58	313.39	102.17	89.23	34.41	0.00
*opie*	327.96	224.42	534.88	444.17	286.28	356.65	0.00	0.00
*giepum*	45.85	26.29	22.95	84.73	20.09	48.38	7.13	13.74
*machiavelli*	12.07	3.73	3.95	3.17	0.00	6.61	0.00	0.00
*wiwa*	10.28	3.33	8.16	9.01	13.90	23.07	5.92	0.00
*uloh*	7.26	0.00	0.00	0.00	0.00	0.00	0.00	0.00
*gudyeg*	7.03	0.00	2.79	0.00	0.00	0.00	0.00	0.00
*eninu*	2.44	2.35	0.00	5.15	6.86	9.71	0.00	0.00
*dijap*	2.27	2.63	0.00	145.58	240.43	159.12	0.00	0.00
*stonor*	1.22	0.00	0.00	6.06	2.71	4.62	0.00	0.00
*anar*	1.03	0.00	0.00	0.00	0.00	0.00	0.00	0.00
*raider*	0.00	3.73	0.00	0.00	11.01	3.71	0.00	0.00
*fourf*	0.00	6.67	11.39	1.48	0.97	1.37	0.00	3.44
*ebel*	0.00	9.42	0.00	0.00	43.14	17.72	0.00	0.00
*maximus*	0.00	0.00	0.00	0.00	0.00	0.00	47.48	15.94
*gudyeg*	0.00	0.00	0.00	0.00	0.00	0.00	3.10	0.00
*gina*	0.00	0.00	0.00	0.00	0.00	0.00	2.79	0.00
*Angela*	0.00	0.00	0.00	0.00	0.00	0.00	1.58	1.66
*osr14*	0.00	0.00	0.00	0.00	0.00	0.00	0.48	0.00
*Ale*	0.00	0.00	0.00	0.00	0.00	0.00	0.30	0.48
*ruda*	0.00	0.00	0.00	0.00	0.00	0.00	0.13	0.00
*Os4*	0.00	0.00	0.00	0.00	0.00	0.00	0.00	7.25
*nuhan*	0.00	0.00	0.00	0.00	0.00	0.00	0.00	6.49
*ekasi*	0.00	0.00	0.00	0.00	0.00	0.00	0.00	2.28
*Unclass.Copia*	70.06	67.44	4.20	35.85	13.57	62.07	63.53	40.61
								
Total	931.50	712.72	1107.90	1048.58	741.14	782.26	166.85	91.90
LTR/*Gypsy*								
*cinful-zeon*	289.37	223.63	583.02	58.70	80.87	65.06	0.00	0.00
*prem1*	220.81	70.62	308.55	170.72	51.08	104.28	0.00	0.00
*flip*	194.03	150.07	351.10	279.59	323.29	166.96	0.00	0.00
*gyma*	146.91	169.35	183.39	213.95	167.51	223.19	4.68	0.00
*xilon-diguus*	142.19	124.76	225.79	45.64	35.02	45.12	0.00	0.00
*tekay*	52.17	23.23	23.41	83.83	14.26	63.59	0.00	32.73
*uwum*	43.27	15.26	18.71	80.17	88.45	64.29	0.00	0.00
*grande*	36.39	80.04	136.62	0.00	86.64	44.80	0.00	0.00
*CRM4*	27.21	17.46	13.73	17.69	18.29	37.73	0.26	0.00
*dagaf*	20.93	47.00	50.30	30.26	55.60	119.78	0.00	0.00
*huck*	14.69	246.19	321.64	1.42	275.99	152.18	0.00	5.37
*doke*	7.55	74.35	78.71	0.00	39.71	46.03	0.00	0.00
*guhis*	4.53	4.12	9.87	1.41	0.94	1.59	0.00	9.71
*puck*	3.99	79.25	23.28	0.00	96.93	90.92	0.00	0.00
*lata*	0.00	0.00	0.00	17.19	0.00	44.18	0.00	0.00
*CRM1*	0.00	0.00	31.73	0.00	2.38	0.00	0.00	0.00
*kuni*	0.00	0.00	0.00	0.00	0.61	0.00	0.00	0.00
*Athila*	0.00	0.00	0.00	0.00	0.00	0.00	56.64	29.53
*Haight*	0.00	0.00	0.00	0.00	0.00	0.00	0.59	0.00
*ahoru*	0.00	0.00	0.00	0.00	0.00	0.00	15.04	0.00
*leviathan*	0.00	0.00	0.00	0.00	0.00	0.00	18.95	66.39
*scDEL*	0.00	0.00	0.00	0.00	0.00	0.00	20.66	0.00
*scTat*	0.00	0.00	0.00	0.00	0.00	0.00	11.10	0.00
*rn_457-190*	0.00	0.00	0.00	0.00	0.00	0.00	13.15	0.00
*evum*	0.00	0.00	0.00	0.00	0.00	0.00	0.00	75.87
*retrosor6*	0.00	0.00	0.00	0.00	0.00	0.00	0.00	181.60
*onap*	0.00	0.00	0.00	0.00	0.00	0.00	0.00	40.13
*deiho*	0.00	0.00	0.00	0.00	0.00	0.00	0.00	13.23
*retrosor1*	0.00	0.00	0.00	0.00	0.00	0.00	0.00	3.17
*keama*	0.00	0.00	0.00	0.00	0.00	0.00	0.00	0.95
*Unclass.Gypsy*	37.05	93.91	29.59	226.53	488.30	492.92	296.70	77.75
Total	1241.09	1419.24	2389.45	1227.10	1825.87	1762.61	437.76	536.43
								
Unclass. Retroelements	93.53	0.00	87.57	159.20	126.08	102.82	0.00	0.00
LINE	2.72	0.00	1.00	2.91	0.00	0.00	1.22	0.94
SINE	0.00	0.00	0.00	0.00	0.00	1.60	0.15	0.00
								
DNA transposons								
DNA.CMC.EnSpm	80.22	61.68	77.51	85.00	88.81	107.27	36.06	48.78
DNA.MULE.MuDR	25.64	4.59	3.20	11.37	15.25	12.62	27.78	6.49
DNA.hAT.Ac	18.51	0.94	0.00	10.56	1.64	1.59	0.74	2.18
Helitron	17.78	9.44	6.75	2.28	0.00	0.00	0.35	1.78
DNA.PIF.Harbinger	14.96	0.00	1.29	4.51	0.00	3.62	2.47	29.57
DNA.TcMar.Stowaway	2.40	0.00	0.00	3.04	0.00	0.00	3.60	6.25
Total	159.90	76.64	88.75	116.75	105.70	125.09	71.00	95.05
Other Repeats								
Satellite	65.84	272.15	754.91	509.75	239.95	379.73	1.76	95.85
Simple repeats	63.14	46.10	0.00	52.94	107.11	13.80	31.42	12.58
rRNA	5.88	61.01	21.74	0.00	34.84	51.58	1.70	14.41
Low complexity	13.86	6.61	5.21	15.97	0.00	0.00	1.71	0.00
Unknown	20.72	3.65	20.38	43.23	0.00	7.41	1.19	4.03
								
Genome proportion	75%	74%	66%	66%	61%	60%	68%	61%

As expected, LTR-retrotransposons are the most abundant repeat in all species. Although the *Zea* species used in this study are diploid and contain the same number of chromosomes, the genome size of *Z. luxurians* (∼4,479 Mb) is nearly double the size of the other two *Zea* species (∼2,600 Mb). From the clustering analysis, *copia* elements were found to contribute approximately 710 Mb, 930 Mb, and 1,110 Mb to the *Z. diploperennis*, *Z. mays* and *Z. luxurians* genomes, respectively. *Gypsy* elements account for ∼1,240 Mb and 1,420 Mb of the *Z. mays* and *Z. diploperennis* genomes, respectively, whereas ∼2,390 Mb of the *Z. luxurians* genome is comprised of *gypsy* elements (Table 1). The greater repeat abundance in *Z. luxurians* correlates with its larger genome size. The *Tripsacum* species have genomes of similar size (∼3,100 Mb) and chromosome number (2n = 36), in which *T. laxum* possesses the smallest genome (2,974 Mb). *Copia* elements occupied ∼740 and 780 Mb in *T. australe* and *T. laxum* genomes, in contrast to 1,050 Mb in the *T. dactyloides* genome. Approximately 1,760 Mb and 1,825 Mb is composed of *gypsy* elements in *T. laxum* and *T. australe*, respectively, whereas the *T. dactyloides* genome contains 1,230 Mb of *gypsy* elements. *Gypsy* elements contributed to more of the genome space (∼53–59%) compared to *copia* (22–28%) in all *Zea* and *Tripsacum* species except for *Z. mays* and *T. dactyloides*, where both *gypsy* and *copia* were nearly equally abundant (Figure 1). *Urelytrum* and *Sorghum* contain ∼167 Mb and 92 Mb of *copia*, and 438 Mb and 536 Mb of *gypsy* elements, respectively.

DNA transposons were found to contribute only 2–6% to the *Zea* and 2–3% to the *Tripsacum* genomes, in contrast to 10–11% in *Urelytrum* and *Sorghum* (Figure 1B). Other groups of repeat elements such as satellite repeats made up a significant fraction of the genome in several species. Approximately 755 Mb of the *Z. luxurians* and 510 Mb of the *T. dactyloides* genomes were occupied by satellite repeats. Although *Urelytrum* and *Sorghum* contain genomes of similar size, the former is composed of only 1.76 Mb of satellite DNA whereas the latter contained ∼100 Mb of satellite DNA.

### Repeat families and their contribution to genome size

From the individual repeat clustering analysis, we identified 24 *copia* and 30 *gypsy* families. Among the 24 *copia* families, *Ji* was the most abundant family in both *Z. mays* (444 Mb) and *Z. diploperennis* (363 Mb), whereas *Opie* was the most abundant in *Z. luxurians* (535 Mb) and in all of the *Tripsacum* species (Table 1). Disproportionately large increases are observed for *Ji* and *Opie* in *Z. mays* and *T. dactyloides*, in agreement with the overall increase of the *copia* superfamily observed for these two species. Interestingly, *Dijap* was estimated at 146-240 Mb in *Tripsacum*, but contributed very little to the genome size of *Zea* (0-2 Mb), indicating lineage-specific accumulation of this family in the *Tripsacum* genus.

Among the *gypsy* families, *Cinful-Zeon*, *Prem1*, *Flip*, *Gyma*, *Huck and Xilon-Diguus* were abundant in both *Zea* and *Tripsacum*. The *Cinful-Zeon* family ranges from 224 - 583 Mb in *Zea* and is positively correlated with genome size, with the greatest abundance in *Z. luxurians*; however, this family contributes only ∼70 Mb to the genomes of the *Tripsacum* species. This is also true for the *Xilon-Diguus* family, with estimates ranging from 125 - 226 Mb in *Zea* and ∼42 Mb in *Tripsacum*, indicating amplification in the lineage leading to *Zea* after divergence from *Tripsacum*. The *Huck* family is estimated at 246 Mb in *Z. diploperennis*, 321 Mb in *Z. luxurians*, 152 Mb in *T. laxum*, and 276 Mb in *T. australe*; however, *Huck* occupies only ∼15 Mb of the *Z. mays* genome and ∼1.4 Mb of the *T. dactyloides* genome. Similarly, elements such as *Doke* and *Puck* were noticeably more abundant in *Z. diploperennis*, *Z. luxurians*, *T. laxum*, and *T. australe*, suggesting independent lineage-specific loss of these families in these *Z. mays* and *T. dactyloides*.

There were 13 *gypsy* families that were specific to *Urelytrum* and/or *Sorghum*. *Athila* and *Leviathan* were relatively abundant (∼19 – 66 Mb) and were identified in both *Urelytrum* and *Sorghum*. Apart from these two families, the remaining 11 *gypsy* families were predominantly present in *Sorghum*, but present in low copy number in *Urelytrum*, or absent altogether. For example, *Retrosor6* is estimated at ∼180 Mb in the genome of *Sorghum* but is completely absent in all other species; however, there is a large number of unclassified *gypsy* elements in *Urelytrum* (Table 1). Although we used a grass specific database to annotate the elements, the majority of this repeat content could not be annotated, suggesting the presence of species-specific repeats and retroelements.

### Comparative clustering analysis

**Figure 2:**
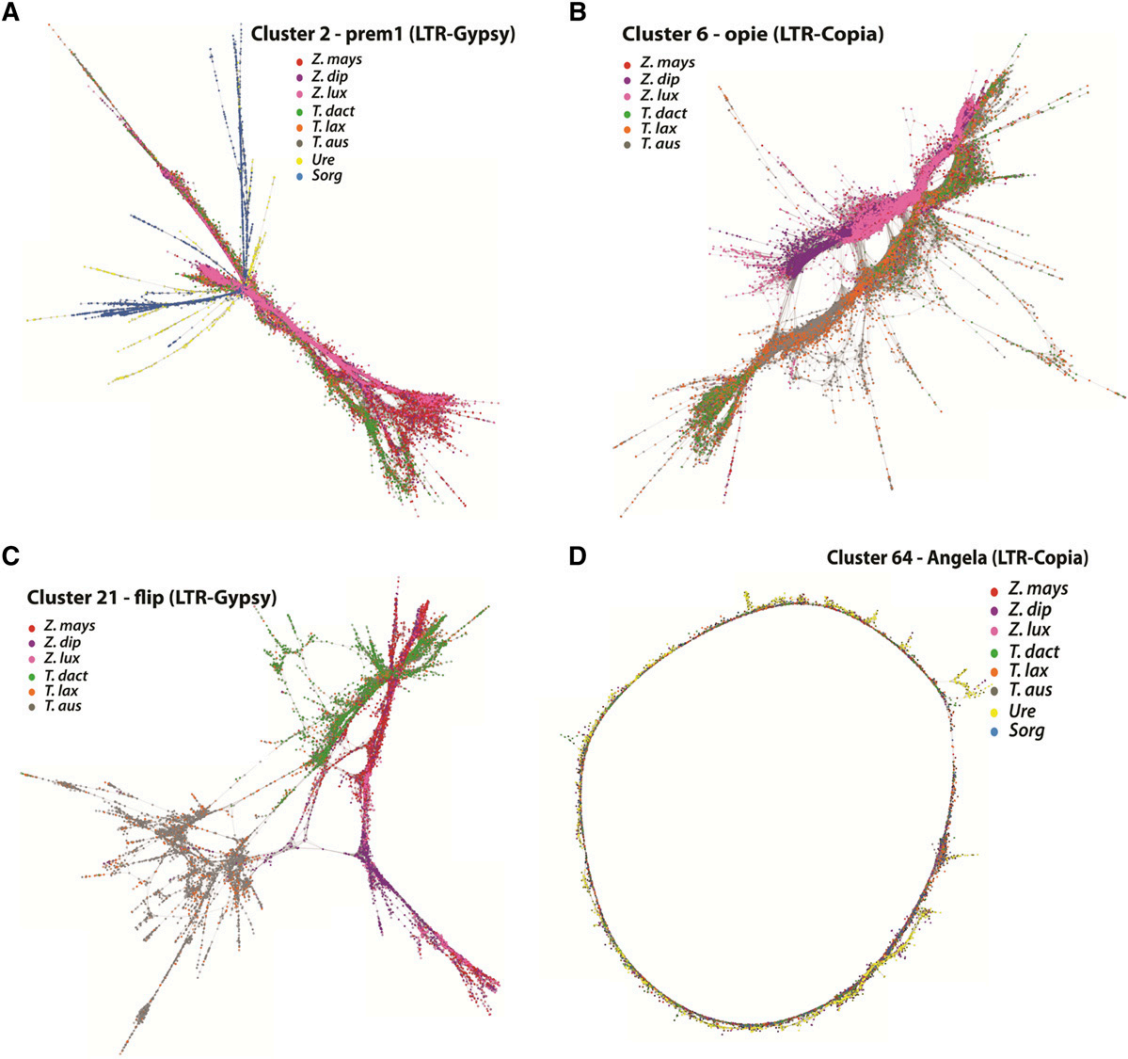
Comparative graph-based clustering. Graphs of individual repeat clusters that are shared between species demonstrating existing sequence variants within species. Highlighted dots represent sequences from individual species and lines connecting the dots represent sequence similarity. Each species is represented with a unique color: Red (*Z. mays*), purple (*Z. diploperennis*), pink *(Z. luxurians*), green (*T. dactyloides*), orange (*T. laxum*), and gray (*T. australe*). A. Cluster 2 shows shared LTR-*gypsy* elements in all genomes, in which sequences of *Zea* and *Tripsacum* are tightly connected with each other and sequences from *Urelytrum* and S*orghum* are peripherally connected, concordant with their evolutionary relationships. B. Cluster 6 (*Opie*, LTR-*copia*) is an example of a lineage-specific repeat family, where sequences are shared between *Zea* and *Tripsacum*; however, there is a clear separation in clustering of both lineages. C. Cluster 21 (*Flip*, LTR-*gypsy*) shows three separate groups in which *Z. mays* and *T. dactyloides* are more similar to one another than either is to their sister species. D. Cluster 64 (*Angela*, LTR-*copia*) is an example of a tightly knitted graph in a linear arrangement shared between all eight species, demonstrating the conserved nature of ancient *Angela* elements across all included taxa.

We performed comparative repeat analysis by simultaneously clustering reads from all eight species in order to identify repeat families that are shared between multiple species and to determine their fate during Andropogoneae evolution, especially during the divergence of *Zea* and *Tripsacum*. This analysis resulted in four major cluster configurations, for which examples are shown in Figure 2A-D. Figure 2A shows an example of a cluster (2: *Prem1*, LTR-*gypsy*) in which the repeat family is common to all species. In this example, most of the reads from *Zea* and *Tripsacum* are tightly clustered, and reads from *Sorghum* and *Urelytrum* are peripherally connected, as would be expected based on their evolutionary relationships. Cluster 6 (*Opie*, LTR*-copia*) is an example of genus-specific repeat accumulation, where sequences are shared between *Zea* and *Tripsacum* but absent in *Urelytrum* and *Sorghum* (Figure 2B). In Cluster 21 (*Flip*, LTR-*gypsy*), the graph indicates three separate groups: one composed primarily of *Z. mays* and *T. dactyloides* [top], one composed primarily of *Z. diploperennis* and *Z. luxurians* [right], and one composed primarily of *T. australe* and *T. laxum* [left]). This type of configuration indicates post-divergence species-specific accumulation independently in *Z. mays* and *T. dactyloides* with minimal transpositional activity in their sister species (Figure 2C). Finally, cluster 64 (*Angela*, LTR-*copia*) is an example of a tightly knitted graph in a circular arrangement shared among all eight species, demonstrating the conserved nature of ancient *Angela* elements across all included taxa (Figure 2D).

**Figure 3:**
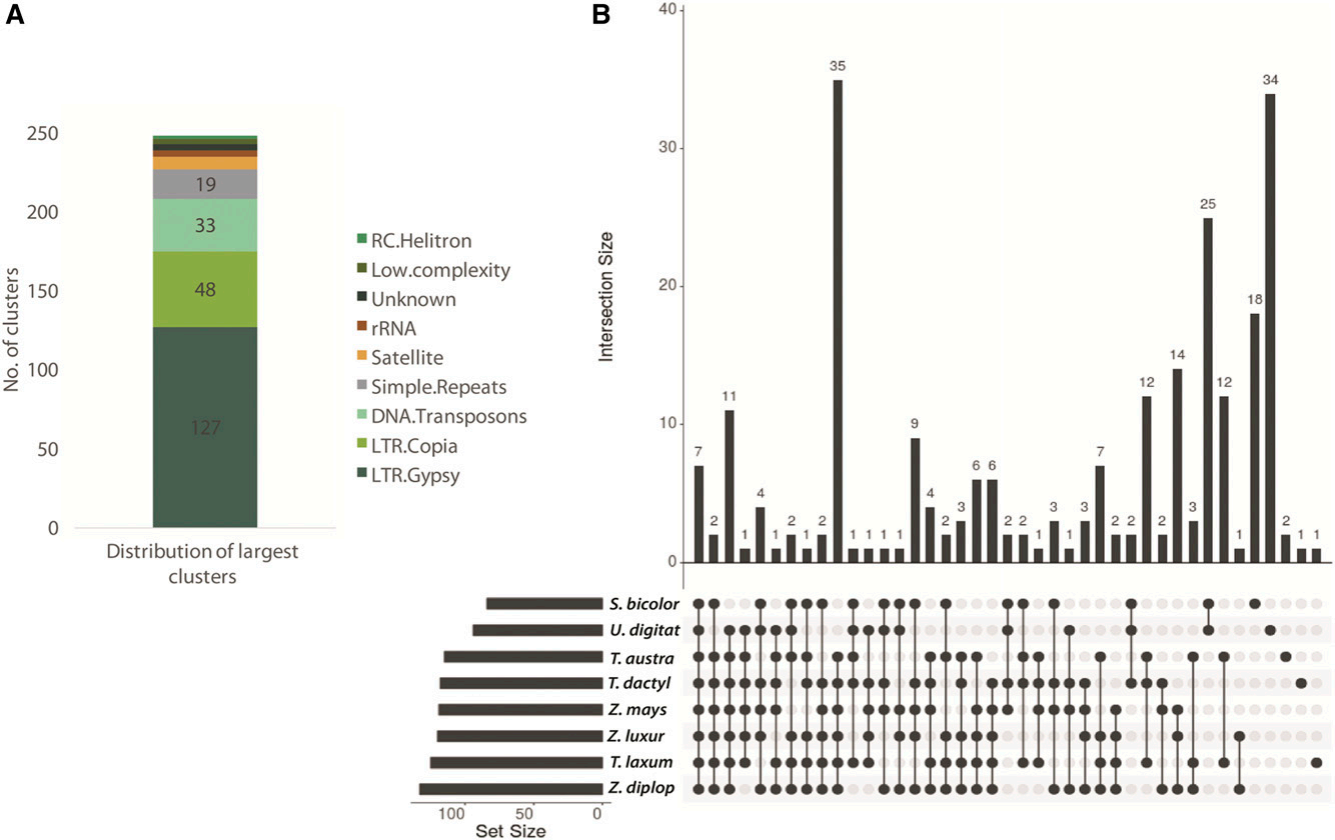
A. Bar graph showing the distribution of the 248 largest clusters with respect to various repeat families. B. UpSet plot showing the interactions of shared repeat clusters among eight species based on sequence similarity of repeats. Each species is represented in one row with filled and empty cells. Each column represents the intersection between each species, *i.e.*, whether the repeat is shared with other species or unique to the respective genome. From left to right, elements shared in all eight species to elements unique to each species are shown. Filled cells connected with black lines indicate that the element is shared with other species. The bars above each intersecting row represent the intersection size.

From a total of two million reads from eight genomes, 248 significant clusters were formed of various sizes and repeat families (Figures 3A and B). On average, ∼81% of the reads from each species clustered with LTR-retrotransposons (127 LTR-*gypsy* and 48 LTR-*copia* clusters). Among the 175 LTR-RT clusters (or families) identified, 85 families were present exclusively in the *Zea-Tripsacum* clade. For all species except *Z. mays* and *T. dactyloides*, the proportion of reads from LTR-*gypsy* families (53%) was higher compared to LTR-*copia* families (28%), whereas *gypsy* and *copia* were nearly equally abundant in *Z. mays* and *T. dactyloides* (Figure 1B). Compared to the other genomes, *Sorghum* contained the smallest proportion (11%) of reads from *copia* families.

Among the 127 *gypsy* clusters, four clusters were shared among all eight species, two clusters were common to *Zea*, *Tripsacum*, and *Urelytrum* (but absent in *Sorghum*), 34 clusters were exclusive to *Zea* and *Tripsacum* species, and 15 clusters were found only in *Urelytrum* and *Sorghum* (Figure S1A). In addition, we observed lineage-specific *gypsy* families: ten in *Zea*, 17 in *Tripsacum*, 21 in *Urelytrum*, and 14 in *Sorghum*. Of the 48 *copia* clusters, only two were common to all species, ten were common to *Zea*, *Tripsacum* and *Urelytrum*, 19 clusters were exclusive to *Zea* and *Tripsacum*, and three were exclusive to *Urelytrum* and *Sorghum* (Figure S1B). Compared to *gypsy* super-families, there were fewer species-specific *copia* families.

### Patterns of insertional bias

Investigation of the genomic distribution of retroelements in *Z. mays* uncovered 2,925 *gypsy* and *copia* elements inserted within 5 kb upstream of genes. The majority of these insertions were within 1-2 kb upstream of genes and were active within the last 1-3 million years (mya) (Figure S2 A and B). According to GO functional enrichment analysis, *gypsy* insertions were associated with genes involved in multicellular organismal process, development processes, and anatomical structure development (Figure S2 C), while *copia* insertions were associated with genes involved in response to abiotic stimulus and small molecule metabolic processes (Figure S2 D). Insertional biases in *Z. luxurians* and *Z. diploperennis* were similar to that of *Z. mays*, with enrichment of *copia* insertions near genes involved in abiotic stress response in addition to cellular, developmental, reproductive, and anatomical structure development (Figure S4, S5-A and B). Similarly, *copia* insertions in *T. dactyloides* were significantly enriched near genes involved in abiotic stress response in addition to processes involved in seed coat development. *Gypsy* insertions in *T. dactyloides* were enriched for starch, glucan and polysaccharide metabolic processes (Figure S3). In contrast, *copia* and *gypsy* insertions in *T. australe* were enriched near metabolic and biosynthetic processes, respectively, with no obvious bias toward genes involved in abiotic stress responses (Figure S4.C and S5.C). No significant enrichment for gene functional categories was found in either *T. laxum* and *U. digitatum*.

In *S. bicolor*, there were 511 *gypsy* and *copia* elements inserted within 5 kb upstream of genes. Most of these insertions were within 2-3 kb upstream of genes and were determined to be more recent insertional events (0-1 mya) compared to maize. In contrast to *Zea* and *Tripsacum*, both *gypsy* and *copia* insertions in *S. bicolor* were associated with genes involved in response to biotic stimulus, endoplasmic reticulum stress, and defense response to other organisms (Figure S6**)**.

### Evolutionary relationships and timing of transposition events

To assess the timing of major transposition events, we constructed maximum likelihood trees using the INT and RT domains (data not shown) of both *gypsy* and *copia* elements. Of the 127 shared *gypsy* clusters, 15 (total of 82 sequences) shared sufficient sequence identity within the integrase domain to allow amino acid sequence alignment. Major repeat families such as *Cinful-xeon*, *Prem1*, *Flip*, *Gyma*, *Xilon-Diguus*, and *Huck* were among these 15 clusters. With a few exceptions, most clades formed as expected in regard to species relationships. The *gypsy* families *Flip* and *Gyma* clustered together. The *Sorghum* and *Urelytrum* sequences from the *Flip* family clustered with *Zea* sequences of *Gyma*, whereas the *Zea* and *Tripsacum* sequences of *Flip* clustered with *Tripsacum* and *Sorghum* of *Gyma*. Several families such as *huck*, *puck*, and *grande* were clustered together with high support, suggesting a recent origin of these families. Clusters such as CL24 (unclassified), *uwum* (CL82) and *guhis* (CL132) also clustered with high sequence similarity.

**Figure 4:**
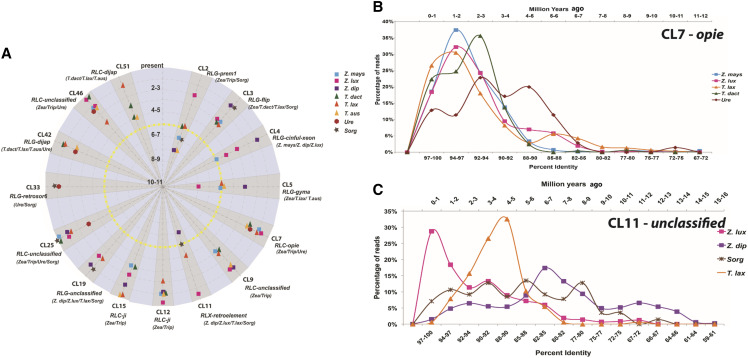
A. Activity of retroelements pre-and post-divergences of *Zea-Tripsacum* (yellow line) for 15 prominent retrotransposon clusters (shaded gray). Concentric circles indicate time scale per million years from 11 mya (center) to present (outer circle). For each cluster, the corresponding repeat family and shared species information is given below each cluster name. Each data point represents the peak activity of that element. B & C display retrotransposon activity of CL7 & CL11 based on percent identity of shared LTR sequences (bottom axis) and the corresponding grass molecular clock (mya) along the top axis. CL11 is absent in *Zea mays* and *Tripsacum dactyloides*.

We employed comparative sequence analyses of LTRs from 15 prominent clusters to estimate the temporal activity of retroelements both pre- and post-divergence of the *Zea-Tripsacum* clade (Figure 4). The clusters chosen for this analysis are composed of the following repeat families: *Prem1*, *Flip*, *Cinful-Xeon*, *Gyma*, *Ji*, *Opie*, *Dijap*, *Retrosor-6*, and several prominent unclassified elements. In Figure 4A, the peak activity of each element per species per cluster is plotted against a TE-specific grass molecular clock (11 mya to present). The approximate timing of the *Zea-Tripsacum* divergence is highlighted in yellow (5-6 mya). *Zea* and *Tripsacum* have experienced post divergence lineage-specific activity for most repeat families. For example, *Ji*, *Opie*, and *Dijap* (CL7, CL12, CL15, CL42, CL19, and CL51) were active within the last three million years for all species in which they are present. The *Opie* element represented in CL7 is shared between *Zea*, *Tripsacum*, and *Urelytrum* and has been active within the last ∼1-3 my indicating that amplification of *Opie* occurred in all three lineages (Figure 4A and 4B). In contrast, the amplification of CL2 (*prem1*) occurred recently only in *Z. luxurians* (2-3 mya) compared to all other species. Although *T. dactyloides* and *T. laxum* experienced increased activity of *Prem1* around the time of divergence, the activity of this element in *Z. mays*, *Z. diploperennis*, *T. australe*, and *Sorghum* dates as an older amplification event. Similarly, the activity of CL5 (*gyma*) is recent in *Z. diploperennis* but older in *Z. luxurians*. Several families were shared only between *Sorghum* and the wild relatives of *Zea (Z. diploperennis*, *Z. luxurians)* and *Tripsacum (T. laxum*, *T. australe)*. Despite their presence, the activity of these families varies among species. For example, the activity of CL11 in *Z. luxurians* is recent (0-1 mya) but is dated as an older event in the other species (Figure 4A and 4C).

## Discussion

The present study evaluates TE dynamics in divergent descendants (*Zea* and *Tripsacum*) of a common allopolyploid ancestor within a phylogenetic framework and in comparison to two diploid relatives (*Urelytrum digitatum* and *Sorghum bicolor*). The comparative analysis of repeat elements from *Zea*, *Tripsacum*, *Urelytrum*, and *Sorghum* provides insight into the contribution of retrotransposons to genome evolution after a shared polyploidization event. Inclusion of additional *Zea* and *Tripsacum* species provided an opportunity to assess genomic variability in repeat content among species within a genus.

As expected, LTR-retrotransposons account for the majority of the repeats in the genomes of all species included in this study. Individual clustering analyses indicate that diverse LTR-retrotransposons contribute to genome size variation in this taxonomic group. Based on our comparative and molecular clock analyses, the majority of retrotransposon families are common to the *Zea – Tripsacum* clade in comparison to their diploid relatives, suggesting the proliferation of retrotransposons after allopolyploidization but before the split between the two genera (Figure 4A). Previous studies have hypothesized an occurrence of retroelement bursts just before the divergence of *Zea* and *Tripsacum* based on maize retroelement activity (Gaut *et al.* 2000, Estep *et al.* 2013). The results for the *Tripsacum* species included in the current analysis support this hypothesis, revealing a high number of shared retrotransposon families between the two genera. For example, *Ji* and *Opie* of the *copia* superfamily have been especially active (2 mya, Figure 4A, >300 Mb, Table 1) in both *Zea* and *Tripsacum*; however, these families contribute little (∼35 Mb) to genome composition in *Urelytrum* and are absent in *Sorghum*. The presence and hyperactivity of these families in *Zea* and *Tripsacum* but not in *Sorghum* suggests amplification after the maize-sorghum split but before the allopolyploidization event leading to the *Zea-Tripsacum* lineage. Similarly, five *gypsy* families (*Cinful-Xeon*, *Prem1*, *Flip*, *Gyma*, and *Huck*) are abundant in the *Zea-Tripsacum* clade but present in low copy numbers in the other lineages. Molecular clock analysis reveals recent activity (1-4 mya) for these families in *Zea* and *Tripsacum*, suggesting amplification after divergence from other taxa (Figure 4A). Conversely, families such as *Athila* and *Leviathan* have accumulated in the *Urelytrum* and *Sorghum* genomes, but are absent in *Zea* and *Tripsacum*, suggesting independent activation of LTR-retrotransposon families in different lineages over short evolutionary time scales.

Additionally, both *Zea* and *Tripsacum* contain lineage-specific families, indicating variation in retroelement amplification in each genus after divergence. Compared to *Zea*, a larger number of *gypsy* and *copia* clusters were unique to *Tripsacum* genomes. Seven *gypsy* families were common to all *Tripsacum* genomes and nine families were shared only between *T. laxum* and *T. australe* (Figure S1 & Figure 4A**)**. The larger number of unique and recently active retroelements (∼1-4 mya) in *Tripsacum* indicates the independent expansion of these families after the *Zea-Tripsacum* divergence. Overall, the abundance and recent activity of these genus-specific LTR-retrotransposons shared only between *Zea* and *Tripsacum* suggests that the activation of these families might be an outcome of shared polyploidization as proposed by the genomic shock hypotheses (McClintock 1984, Comai *et al.* 2003).

Surprisingly, clustering analyses suggest that *Z. mays* and *T. dactyloides* share greater similarity in TE composition and abundance for some retrotransposon families than either does to the other members of their respective genera. Examples of noticeably increased abundance in these two species include the families *Ji*, *Flip*, and *Opie*. Conversely, we also found significantly decreased copy numbers of *Huck*, *Doke*, and *Puck*. Also, a greater number of reads from both genomes are derived from the *copia* superfamily, indicating independent expansion of *copia* clades in both lineages. For a few shared *copia* clusters, the peak of activity in *Z. mays* overlaps with *T. dactyloides*, suggesting both species experienced *copia* activity during a similar time period (Figure 4A**)**. Considering the independent evolution of both species post divergence and the role of artificial selection in maize domestication, the similarity in composition and activity of *copia* elements in *Z. mays* and *T. dactyloides* suggests that natural and artificial selection have influenced TE amplification and accumulation similarly in both lineages. Although the precise cause of such species-specific activity is unclear, we propose that the observed patterns in TE abundance and accumulation may be related to adaptation to temperate climates. Indeed, recent evidence shows parallels in protein sequence evolution between natural and artificial selection in *T. dactyloides* and maize during their adaptation to temperate climates (Yan *et al.* 2019).

Considering the intensity of retroelement accumulation in maize as reported in other studies, it is likely that these elements have been active during maize domestication and improvement. Studies demonstrating TE involvement in plant domestication predominantly show that insertions near functional genes play a role in plant function and/or development. Well-known examples include *Hopscotch* involvement in apical dominance in maize (Studer *et al.*, 2011), *Gret1* in berry color variation in *Vitis vinifera* (Cadle-Davidson and Owens 2008), and LTR-mediated control of the blood orange phenotype (Butelli *et al.* 2012). Therefore, we explored the frequency of LTR-retrotransposon insertions near genes. In *Z. mays*, *Z. diploperennis*, *and Z. luxurians*, *gypsy* insertions were enriched near genes involved in multicellular, developmental, and metabolic processes whereas *copia* insertions were enriched near abiotic stress-associated and defense response genes (Figure S2-S5). In contrast with *Z. mays*, *copia* insertions in *Z. diploperennis* and *Z. luxurians* were also enriched near genes involved in developmental and metabolic processes. Indeed, *Z. diploperennis* and *Z. luxurians* shared similar GO enrichment profiles. *T. dactyloides* also demonstrated enrichment of *copia* insertions near abiotic stress-associated genes, but *copias* were also enriched near genes involved in development, while *gypsys* were enriched near genes involved in metabolic function, in contrast to findings in *Zea*. *T. australe* and *T. laxum* did not show enrichment for insertions near genes involved in the abiotic stress response. These results demonstrate that, with the exception of the insertional distributions in *Z. diploperennis* and *Z. luxurians*, TE insertional mutagenesis after polyploidization and divergence has been dynamic and lineage-specific.

The commonality of *copia* insertions near abiotic stress associated-genes in *Zea* and in *T. dactyloides* suggests that they may play a role in molding the plant response to environmental stress. Given our findings that *copias* have been particularly active in *Z. mays* and *T. dactyloides*, and that these insertions are predominantly near abiotic stress-associated genes, these results provide further support for a role during adaptation to temperate climates. Such insertional proximity provides the potential for TEs to affect the function of neighboring genes (Makarevitch *et al.* 2015, Cao *et al.* 2016, Pietzenuk *et al.* 2016). For example, the tobacco Tnt1 and the rice Tos17 *copia* elements were found near stress-related genes, and the expression of these elements was linked with the biological responses of the plant to external stress (Grandbastien *et al.* 1997, Miyao *et al.* 2003, Le *et al.* 2007).

## Conclusion

In this study, we provide insight into interspecific TE diversity and its contribution to genome evolution in related members of Andropogoneae that have undergone a shared polyploidization event. By including multiple accessions of two divergent species (*Zea* and *Tripsacum*) originating from a common allopolyploid ancestor, in addition to close diploid relatives (*Urelytrum digitatum* and *Sorghum bicolor*), we described LTR-retrotransposon diversity with respect to the hybridization and genome doubling process. Though the genome size of *Urelytrum* is similar to that of *Sorghum*, the repeat composition of *Urelytrum* is more like that of *Zea* and *Tripsacum*. Similarities in the proportion of the *copia* superfamily and satellite DNA in *Zea-Tripsacum-Urelytrum* suggests that *Urelytrum* or a close relative may have played an ancestral role in the origin of the *Zea-Tripsacum* lineages, supporting the hypothesis proposed by McKain *et al.* (2018). The genomic distribution of retroelements near protein coding genes of similar function in addition to the expansion of the *copia* superfamily exclusively in *Z. mays* and *T. dactyloides*, suggests amplification (and possibly participation) of new *copia* insertions during adaptation to temperate environments. Indeed, many of the *copia* insertions are near genes involved in abiotic stress and defense factors. Therefore, the *cis*-regulatory effects of TE insertions near genes may have influenced the evolution of *Z. mays* and *T. dactyloides* during both adaptation and domestication.
